# Supplementary material for: Nonpathogenic Pseudomonas syringae derivatives and its metabolites trigger the plant “cry for help” response to assemble disease suppressing and growth promoting rhizomicrobiome
Source: Nat Commun. 2024 Mar 1;15:1907. doi: 10.1038/s41467-024-46254-3 (PMC10907681; doi:10.1038/s41467-024-46254-3)
Supplement: Supplementary file 1 — Supplementary Information [file 41467_2024_46254_MOESM1_ESM.pdf]

Supplementary files

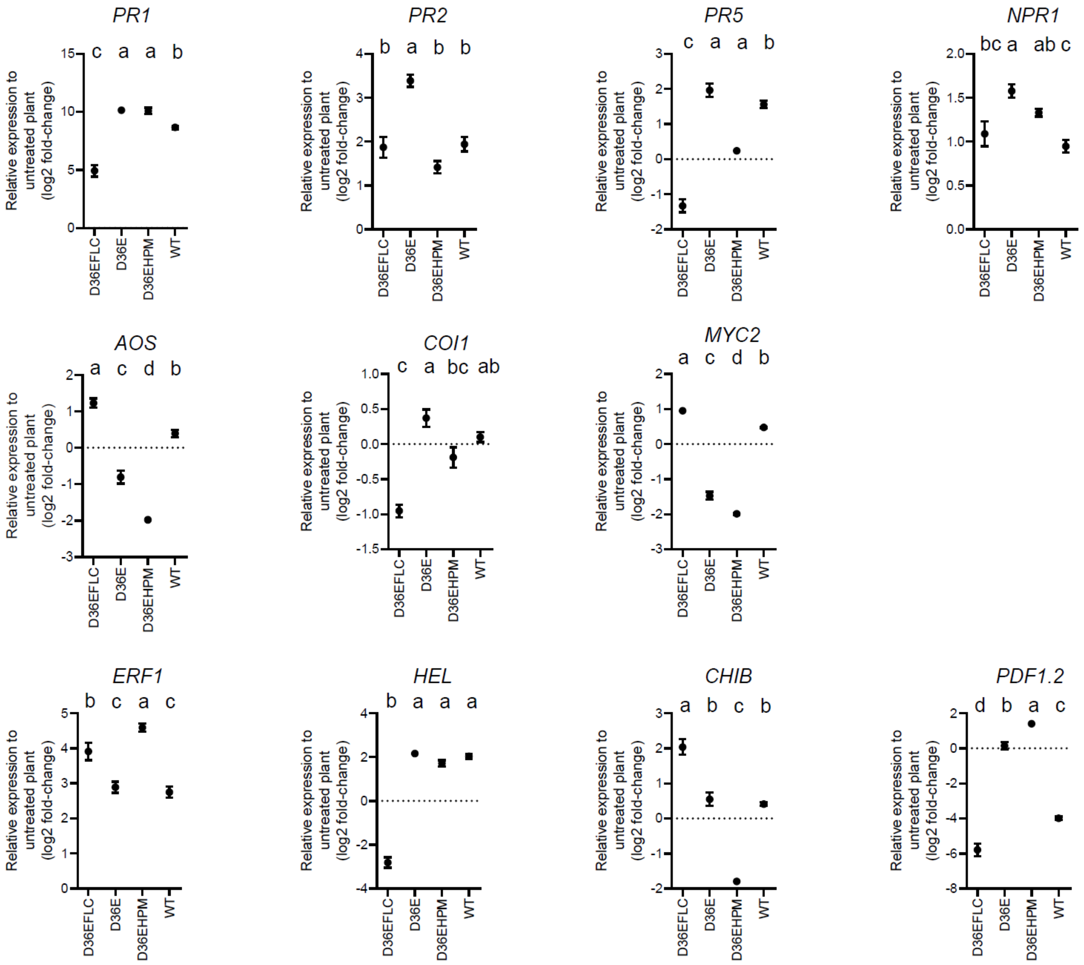

Supplemental Figure 1. Expression of genes related to phytohormone signaling of Arabidopsis in response to wild-type DC3000 and the derivatives by qPCR. Expression of *ACTIN2* was included as the internal reference. Data are presented as relative expression to untreated plant as mean values +/- SEM. The different letters indicate significant differences (α=0.05, n=6 samples) according to ANOVA based on Duncan's multiple range test, P<0.001 for all panels.

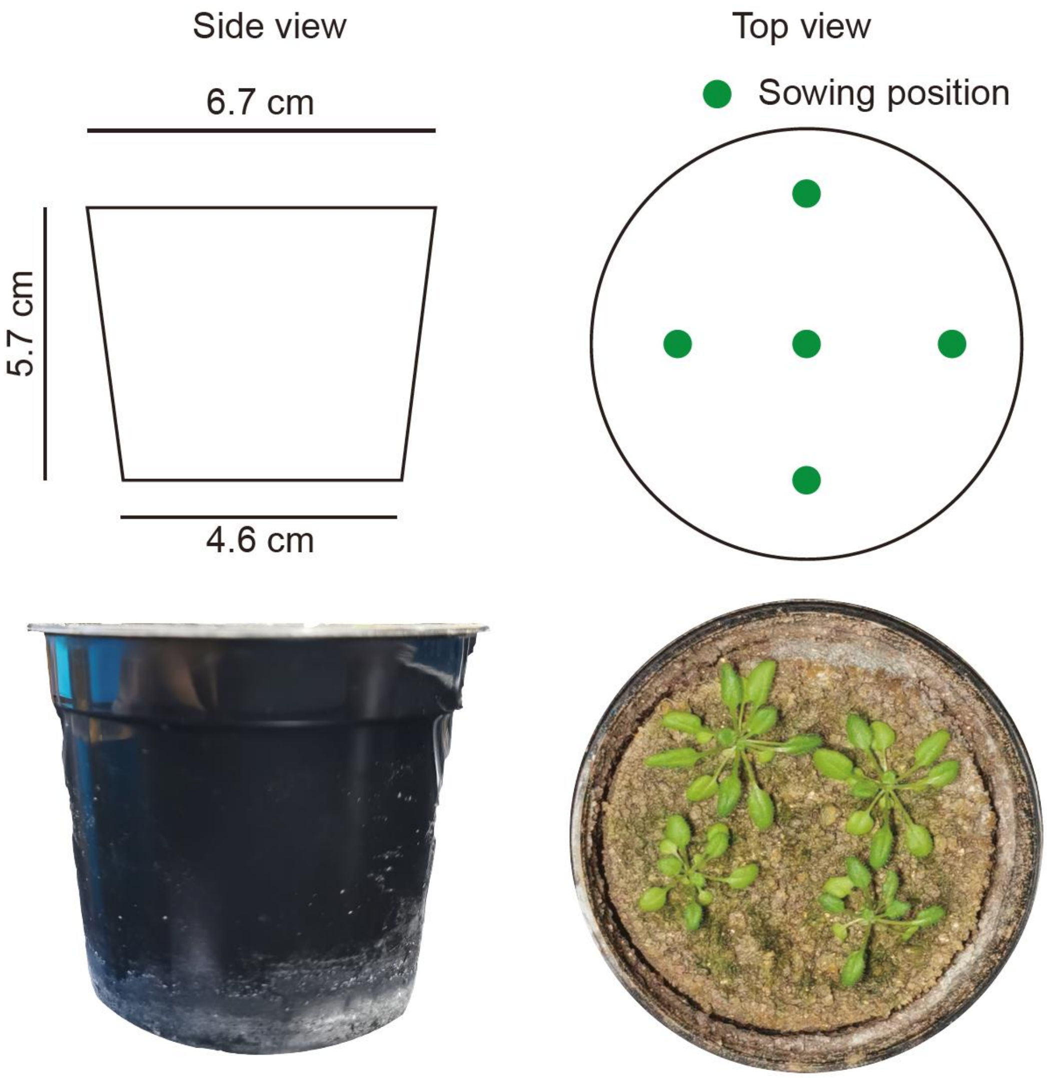

Supplementary Figure 2. Overview of the pot experiment.

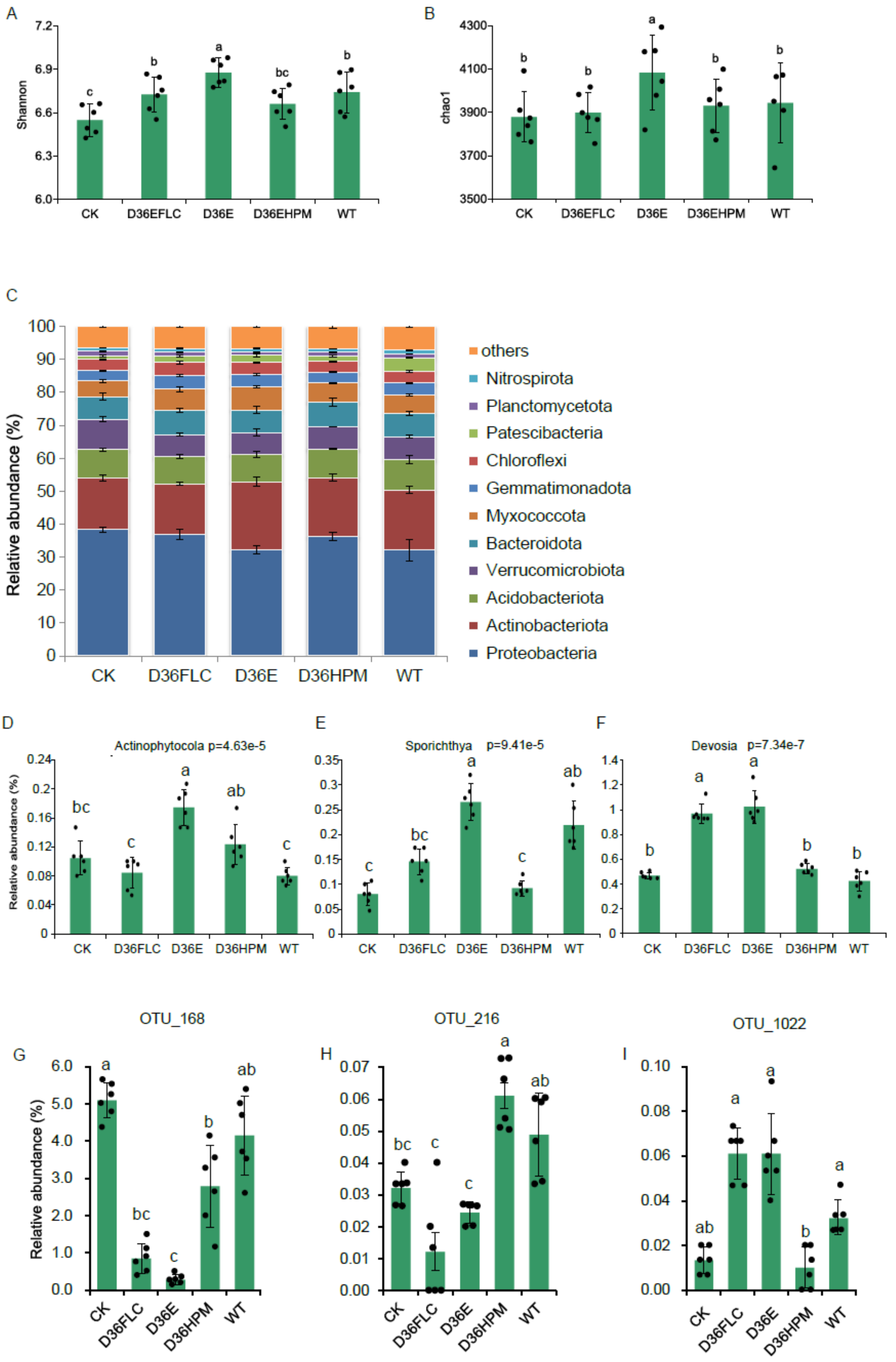

Supplementary Figure 3. Composition of the rhizosphere microbiome of Arabidopsis grown in soil with legacy. (A-B) Shannon and Chao1 of the rhizosphere microbiome from

plants grown in soil containing legacy. The different letters indicate significant differences ($\alpha=0.05$, n=6 independent samples, $P=0.001$ for A, $P=0.018$ for B) according to ANOVA based on Duncan's multiple range test. (C) Relative abundance of the phyla. Data are presented as mean values +/- SEM (n=6 independent samples). (D-F) Relative abundance of the genera Sporichthya, Actinophytocola and Devosia. Data are presented as mean values +/- SEM (n=6 independent samples). The different letters indicate significant differences according to two-sided ANOVA based on Kruskal-Wallis test ($\alpha=0.05$, n=6 independent samples, $P<0.001$). (G-I) Relative abundance of OTU_168, OTU _216 and OTU _1022. Data are presented as mean values +/- SEM (n=6 independent samples). The different letters indicate significant differences according to two-sided ANOVA based on Kruskal-Wallis test ($\alpha=0.05$, n=6 independent samples, $P<0.001$).

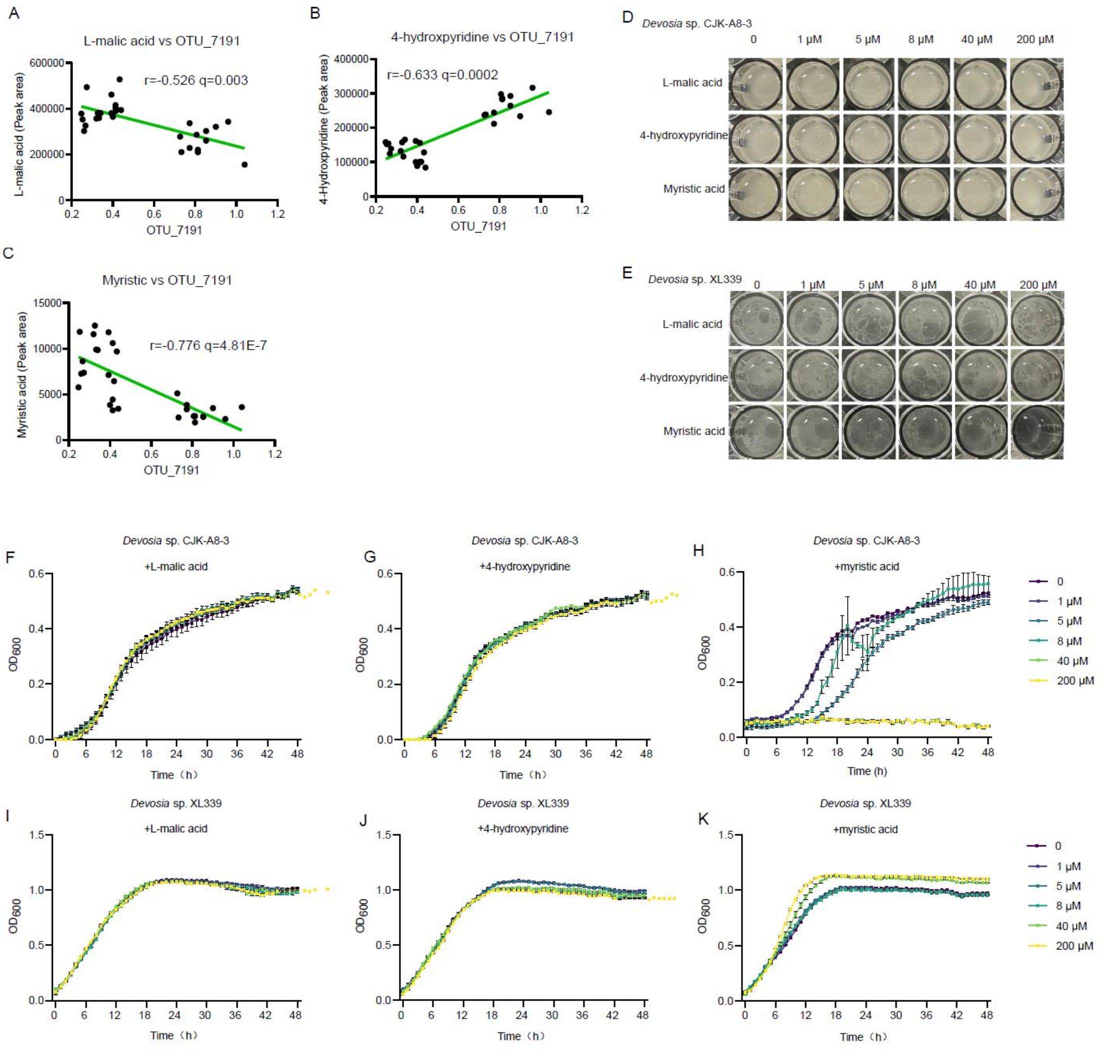

Supplementary Figure 4. Reduced myristic acid in root exudates of D36E- and D36EFLC-treated plants contributes to *Devosia* enrichment. (A-C) Linear regression of the abundance of OTU7191 to the abundance of L-malic acid (A), 4-hydroxypridine (B) and myristic acid (C) in root exudates. Spearman correlation was performed, and the q value was calculated based on BH. (D-E) Biofilm formation test for *Devosia* sp. CJK-A8-3 (D) and *Devosia* sp. XL339 (E) in MSgg medium. L-malic acid, 4-hydroxypridine and myristic acid were added to the medium with a concentration gradient. Images were taken at 72-h post inoculation. n=4 independent experiments. (F-K) Growth curve for *Devosia* sp. CJK-A8-3 (F-H) and *Devosia* sp. XL339 (I-L) with L-malic acid (G and J), 4-hydroxypridine (H and K) and myristic acid (I and L) in TSB medium. The OD_{600} was recorded every hour throughout the growth with a Biocreen C growth detector system. For F-K, data are presented as mean values +/- SEM (n=6 independent experiments).

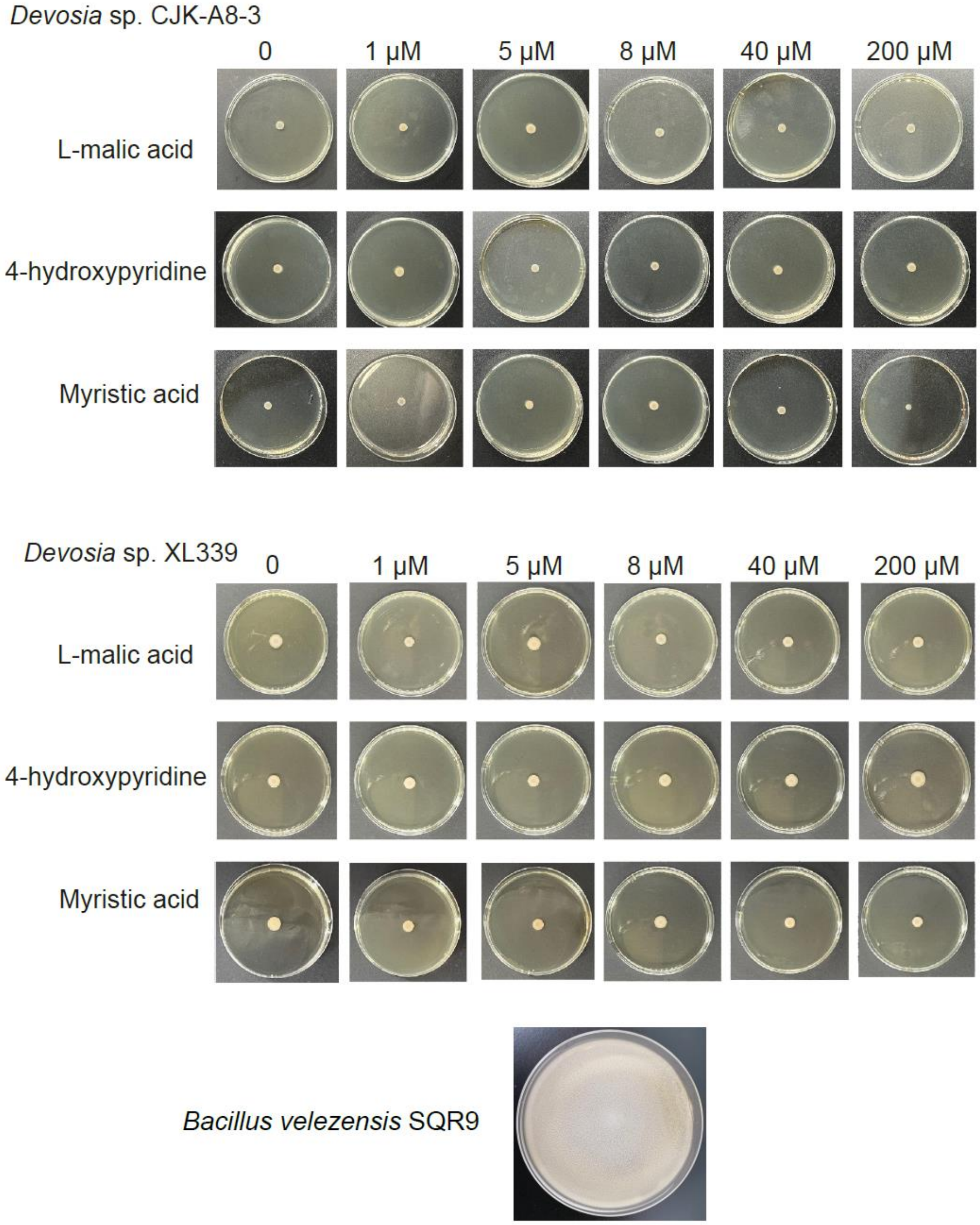

Supplementary Figure 5. The motility of *Devosia* sp. CJK-A8-3 and *Devosia* sp. XL339 on swarming plates. Images were taken at 16-h post inoculation. The motile bacterium *Bacillus velezensis* SQR9 was used as a positive control. Four replicates were included for each treatment.

Supplementary Table 1. Physical and chemical characteristics of the soil used in this study

Soil properties	Values
pH	6.67 ± 0.08
Organic matter (g/kg)	29.47 ± 0.81
Total N (g/kg)	2.72 ± 0.07
Total P (g/kg)	0.52 ± 0.01
Total K (g/kg)	23.05 ± 0.40
Available N (mg/kg)	233.11 ± 2.95
Available P (mg/kg)	3.43 ± 0.14
Available K (mg/kg)	188.03 ± 22.59
Electrical conductivity (µs/cm)	84 ± 2.40
Cation exchange capacity (cmol/kg)	26.67 ± 3.24

Supplementary Table 2. OTUs significantly altered in rhizosphere of plant grown in SL-D36E and SL-D35EFLC

OTUID	Kingdom	Phylum	Class	Order	Family	Genus	Species
OTU_7191	Bacteria	Proteobacteria	Alphaproteobacteria	Rhizobiales	Devosiaceae	Devosia	Unassigned
OTU_216	Bacteria	Bdellovibrionota	Oligoflexia	0319-6G20	Unassigned	Unassigned	Unassigned
OTU_1022	Bacteria	Actinobacteriota	Thermoleophilia	Solirubrobacterales	Solirubrobacteraceae	Unassigned	Unassigned
OTU_168	Bacteria	Proteobacteria	Gammaproteobacteria	Burkholderiales	Nitrosomonadaceae	Ellin6067	Unassigned

Supplementary Table 3 Strains used in this study

Strain	Description	Reference
WT	Wild-type *P. syringae pv. tomato* DC3000; Rif^r	[1]
D36E	DC3000 that deleted all 36 effectors; Rif^r; Sp^r	[2]
D36EFLC	D36EΔ*fliC*; Rif^r; Sp^r	[3]
D36EHPM	D36E-Tn7-ShcM-HopM1-HA; Rif^r, Sp^r,Km^r	[3]
D36EavrRpt2	D36E-avrRpt2; Rif^r, Km^r	[4]

Supplementary Table 4. Primers used in this study

Primer Name	Sequence(5'to3')
ACTIN2-F	CCTGCCATGTATGTTGCCATT
ACTIN2-R	AATCGAGCACAATACCGGTTGT
PR1-F	AGGTGCTCTTGTTCTTCCCT
PR1-R	ACCCCAGGCTAAGTTTTCCC
PR2-F	TGGTGTCAGATTCCGGTACA
PR2-R	TCATCCCTGAACCTTCCTTG
PR5-F	GGAACAATTGCCCTACCACC
PR5-R	GCCGTTACATCTTAGACCGC
NPR1-F	ACCGATAACACCGACTCCTC
NPR1-R	GCACCGGTGGAAAGAAACTT
AOS-F	TGAGTTTGTGCCGGAGAGAT
AOS-R	ATCACAAACAACCTCGCCAC
COI1-F	TCAAATCGGTGCACTTCCGA
COI1-R	ACCTCAAAAGCATCGAGCCA
MYC2-F	ATAAATCTCCAGCTCCGCCG
MYC2-R	AAGCGTTTGCAACGGGTAAC
ERF1-F	AGGATGGTTGTTCTCCGGTT
ERF1-R	AGACCCCAAAAGCTCCTCAA
HEL-F	ATCTGCTGCAGTCAGTACGG
HEL-R	TGAGCTCATTGCCACAGTCG
CHIB-F	GCTTCAGACTACTGTGAACC
CHIB-R	TCCACCGTTAATGATGTTCG
PDF1.2-F	CACCCTTATCTTCGCTGCTC
PDF1.2-R	GCACAACTTCTGTGCTTCCA

Supplementary code

OTU PCA code:
The script is run by R (v 3.6.0) for PCoA of microbiome.

R (v 3.6.0) and R studio (2022.02.3 Build 492) should be installed. (Install time: as instructed for R and R studio)

```
library(ade4)
library(ggplot2)
library(RColorBrewer)
library(vegan)
library(ggsci)
library(readxl)
library(readr)
Group <- read_delim("Group.txt", delim = "\t",escape_double = FALSE, trim_ws = TRUE)
group<-as.factor(Group$Group)
group<-factor(Group$Group,levels=c("CK","D36EFLC","D36E","D36EHPM","WT"))
OTU_TABLE <- read_excel("Test data.xlsx")
Group <- read_delim("Group.txt", delim = "\t",escape_double = FALSE, trim_ws = TRUE)
OTU<-t(OTU_TABLE[,-1])
OTU.dist<-vegdist(OTU,method='euclidean')
pcoa<- dudi.pco(OTU.dist, scan = FALSE,nf=3)
pcoa_eig <- (pcoa$eig)[1:2] / sum(pcoa$eig)
sample_site <- data.frame({pcoa$li})[1:2]
sample_site$names<-rownames(sample_site)
names(sample_site)[1:2]<-c('PCoA1','PCoA2')
sample_site$level<-group

pcoa_plot <- ggplot(sample_site, aes(PCoA1, PCoA2,color=level))+geom_vline(xintercept =
0, color = 'black', size = 0.4)+geom_hline(yintercept = 0, color = 'black', size =
0.4)+geom_point(size = 1.5)+ theme(panel.grid = element_line(color = 'black', linetype = 2,
size = 0.1),panel.background = element_rect(color = 'black', fill =
'transparent'),legend.title=element_blank())+labs(x = paste('PCoA1: ', round(100 *
pcoa_eig[1], 2), '%'), y = paste('PCoA2: ', round(100 * pcoa_eig[2], 2), '%'))
+scale_color_npg()
```

Root exudates PCA code:

The script is run by R (v 3.6.0) for PCoA of root exudates composition.

R (v 3.6.0) and R studio (2022.02.3 Build 492) should be installed. (Install time: as instructed for R and R studio)

```
library(ade4)
library(ggplot2)
library(RColorBrewer)
library(vegan)
library(ggsci)
library(readxl)
library(readr)

Group <- read_delim("Group.txt", delim = "\t",escape_double = FALSE, trim_ws = TRUE)
group<-as.factor(Group$Group)
group<-factor(Group$Group,levels=c("CK","D36EFLC","D36E","D36EHPM","WT"))
Compounds_for_pcoa <- read_excel("Test data.xlsx")
RE<-Compounds_for_pcoa
Roote<-t(RE[,-1])
Roote.dist<-vegdist(Roote,method='euclidean')
pcoa<- dudi.pco(Roote.dist, scan = FALSE,nf=3)
pcoa_eig <- (pcoa$eig)[1:2] / sum(pcoa$eig)
sample_site <- data.frame({pcoa$li})[1:2]
sample_site$names<-rownames(sample_site)
names(sample_site)[1:2]<-c('PCoA1','PCoA2')
sample_site$level<-group
pcoa_plot <- ggplot(sample_site, aes(PCoA1, PCoA2,color=level))+geom_vline(xintercept =
0, color = 'black', size = 0.4)+geom_hline(yintercept = 0, color = 'black', size =
0.4)+geom_point(size = 1.5)+ theme(panel.grid = element_line(color = 'black', linetype = 2,
size = 0.1),panel.background = element_rect(color = 'black', fill =
'transparent'),legend.title=element_blank())+labs(x = paste('PCoA1: ', round(100 *
pcoa_eig[1], 2), '%'), y = paste('PCoA2: ', round(100 * pcoa_eig[2], 2), '%'))
+scale_color_npg()
```

References

1. Cuppels, D. A. Generation and characterization of Tn5 insertion mutations in *Pseudomonas syringae* pv. *tomato*. *Applied and Environmental Microbiology* vol. 51 323–327 (1986).
2. Wei, H. L. *et al. Pseudomonas syringae* pv. *tomato* DC3000 type III secretion effector polymutants reveal an interplay between HopAD1 and AvrPtoB. *Cell Host Microbe* **17**, 752–762 (2015).
3. Wei, H. L., Zhang, W. & Collmer, A. Modular study of the type III effector repertoire in *Pseudomonas syringae* pv. *tomato* DC3000 reveals a matrix of effector interplay in pathogenesis. *Cell Rep.* **23**, 1630–1638 (2018).
4. Yuan, M. *et al.* Pattern-recognition receptors are required for NLR-mediated plant immunity. *Nature* **592**, 105–109 (2021).
