# Supplementary material for: Nonpathogenic Pseudomonas syringae derivatives and its metabolites trigger the plant “cry for help” response to assemble disease suppressing and growth promoting rhizomicrobiome
Source: Nat Commun. 2024 Mar 1;15:1907. doi: 10.1038/s41467-024-46254-3 (PMC10907681; doi:10.1038/s41467-024-46254-3)
Supplement: Supplementary file 2 — Reporting Summary [file 41467_2024_46254_MOESM2_ESM.pdf]

Corresponding author(s): Yunpeng Liu, Weibing Xun, Ruifu Zhang

Last updated by author(s): Feb 14, 2024

Reporting Summary

Nature Portfolio wishes to improve the reproducibility of the work that we publish. This form provides structure for consistency and transparency in reporting. For further information on Nature Portfolio policies, see our Editorial Policies and the Editorial Policy Checklist.

Statistics

For all statistical analyses, confirm that the following items are present in the figure legend, table legend, main text, or Methods section.

n/a	Confirmed	
☐	☒	The exact sample size (*n*) for each experimental group/condition, given as a discrete number and unit of measurement
☐	☒	A statement on whether measurements were taken from distinct samples or whether the same sample was measured repeatedly
☐	☒	The statistical test(s) used AND whether they are one- or two-sided<br>*Only common tests should be described solely by name; describe more complex techniques in the Methods section.*
☒	☐	A description of all covariates tested
☐	☒	A description of any assumptions or corrections, such as tests of normality and adjustment for multiple comparisons
☐	☒	A full description of the statistical parameters including central tendency (e.g. means) or other basic estimates (e.g. regression coefficient) AND variation (e.g. standard deviation) or associated estimates of uncertainty (e.g. confidence intervals)
☐	☒	For null hypothesis testing, the test statistic (e.g. *F*, *t*, *r*) with confidence intervals, effect sizes, degrees of freedom and *P* value noted<br>*Give P values as exact values whenever suitable.*
☒	☐	For Bayesian analysis, information on the choice of priors and Markov chain Monte Carlo settings
☒	☐	For hierarchical and complex designs, identification of the appropriate level for tests and full reporting of outcomes
☒	☐	Estimates of effect sizes (e.g. Cohen's *d*, Pearson's *r*), indicating how they were calculated

Our web collection on statistics for biologists contains articles on many of the points above.

Software and code

Policy information about availability of computer code

Data collection	Deep sequencing was performed on the MiSeq platform at Allwegene Company (Beijing). Root exudates composition were analyzed by an Agilent 7890 gas chromatograph system coupled to a Pegasus HT time-of-flight mass spectrometer. OD600 measurement was performed using the Bioscreen C system.
Data analysis	After sequencing was performed, image analysis, base calling and error estimation were performed using Illumina Analysis Pipeline version 2.6. The qualified reads were separated using the sample-specific barcode sequences and trimmed with Illumina Analysis Pipeline version 2.6. Then, the dataset was analyzed using QIIME. The Ribosomal Database Project (RDP) Classifier tool was used to classify all the sequences into different taxonomic groups.Chroma TOF 4.3X software (LECO Corporation) and the LECO-Fiehn Rtx5 database were used for extracting the raw peaks, filtering the data baselines, calibrating the baseline, aligning the peaks, analyzing the deconvolution, identifying the peaks and integrating the peak areas. R (version 3.6) and SPSS (v27)was used for statistical analysis.

For manuscripts utilizing custom algorithms or software that are central to the research but not yet described in published literature, software must be made available to editors and reviewers. We strongly encourage code deposition in a community repository (e.g. GitHub). See the Nature Portfolio guidelines for submitting code & software for further information.

Data

Policy information about availability of data

All manuscripts must include a data availability statement. This statement should provide the following information, where applicable:

- Accession codes, unique identifiers, or web links for publicly available datasets
- A description of any restrictions on data availability
- For clinical datasets or third party data, please ensure that the statement adheres to our policy

Source data are provided with this paper. The 16S raw data of rhizosphere microbiome generated have been deposited in the NCBI database under accession code PRJNA803271 (www.ncbi.nlm.nih.gov/bioproject/?term=PRJNA803271%20). The 16S rRNA sequence of Devosia sp. strain CJK-A8-3 and Devosia sp. strain XL339 have been deposited in GenBank with the accession code PP325837 and PP325838, respectively (https://www.ncbi.nlm.nih.gov/nuccore/?term=PP325837:PP325838). The metabolomics mass spectrometry raw data are only available for request because there are other uses of the mass spectrometry raw data.

Research involving human participants, their data, or biological material

Policy information about studies with human participants or human data. See also policy information about sex, gender (identity/presentation), and sexual orientation and race, ethnicity and racism.

Reporting on sex and gender	Not applicable
Reporting on race, ethnicity, or other socially relevant groupings	Not applicable
Population characteristics	Not applicable
Recruitment	Not applicable
Ethics oversight	Not applicable

Note that full information on the approval of the study protocol must also be provided in the manuscript.

Field-specific reporting

Please select the one below that is the best fit for your research. If you are not sure, read the appropriate sections before making your selection.

- [x] Life sciences
- [ ] Behavioural & social sciences
- [ ] Ecological, evolutionary & environmental sciences

For a reference copy of the document with all sections, see nature.com/documents/nr-reporting-summary-flat.pdf

Life sciences study design

All studies must disclose on these points even when the disclosure is negative.

Sample size	We did not perform a calculation for sample size, but we made sure that a sufficient number of replicates were taken following previous experience with these types of experimental data(DOI:10.1038/NMICROBIO.2016.183). To ensure the reproducibility, some experiments were performed for several times with relevant independent replicates for each time of experiment, the detailed times of experiment were stated in the paper.
Data exclusions	No data was excluded.
Replication	For reactive oxygen species measurement, six replicates were included for each treatment, and the entire ROS experiment was repeated twice. For plant hypersensitive response assays, this experiment was repeated twice, with 9 replicates for each treatment. For the root exudates composition analyzing and microbiome investigation, 6 replicates were included for each treatment. For biofilm formation experiment, each treatment included 4 replicates. For measuring the growth curve, 6 replicates were included for each treatment. All attempts at replication was successful for all the experiment. For germination experiment, 20 replicates (Figure 3 and Figure 5)or 7 replicates (Figure 4) were included. For plant growth experiments, all plants grown in the pots were included as replicates, the detailed number is shown in figure legends. For pathogen colonization experiments, 20 replicates were included. All attempts at replication was successful for all the experiment.
Randomization	For all the experiments grown Arabidopsis in pots, all the pots were randomly placed.
Blinding	Blinding is not relevant for our study that investigated interactions between bacteria with their host plant.

Reporting for specific materials, systems and methods

We require information from authors about some types of materials, experimental systems and methods used in many studies. Here, indicate whether each material, system or method listed is relevant to your study. If you are not sure if a list item applies to your research, read the appropriate section before selecting a response.

Materials & experimental systems

n/a	Involved in the study
☒	☐ Antibodies
☒	☐ Eukaryotic cell lines
☒	☐ Palaeontology and archaeology
☒	☐ Animals and other organisms
☒	☐ Clinical data
☒	☐ Dual use research of concern
☒	☐ Plants

Methods

n/a	Involved in the study
☒	☐ ChIP-seq
☒	☐ Flow cytometry
☒	☐ MRI-based neuroimaging

Plants

Seed stocks

Arabidopsis thaliana ecotype Columbia (Col-0) was used in this study.

Novel plant genotypes

Not applicable

Authentication

Not applicable
