# Supplementary material for: Nonpathogenic Pseudomonas syringae derivatives and its metabolites trigger the plant “cry for help” response to assemble disease suppressing and growth promoting rhizomicrobiome
Source: Nat Commun. 2024 Mar 1;15:1907. doi: 10.1038/s41467-024-46254-3 (PMC10907681; doi:10.1038/s41467-024-46254-3)
Supplement: Supplementary file 4 — Description of Additional Supplementary Files [file 41467_2024_46254_MOESM4_ESM.pdf]

Description of Additional Supplementary Files

File Name: Supplementary Data 1

Description: Composition of root exudates.

File Name: Supplementary Data 2

Description: Correlation between OTU_7191 and compounds in root exudates.

File Name: Supplementary Data 3

Description: Raw OTU counts before rarefication.
