# Supplementary material for: Nonpathogenic Pseudomonas syringae derivatives and its metabolites trigger the plant “cry for help” response to assemble disease suppressing and growth promoting rhizomicrobiome
Source: Nat Commun. 2024 Mar 1;15:1907. doi: 10.1038/s41467-024-46254-3 (PMC10907681; doi:10.1038/s41467-024-46254-3)
Supplement: Supplementary file 8 — Source data [file 41467_2024_46254_MOESM8_ESM.zip › SourceData_Fig1_images.docx]

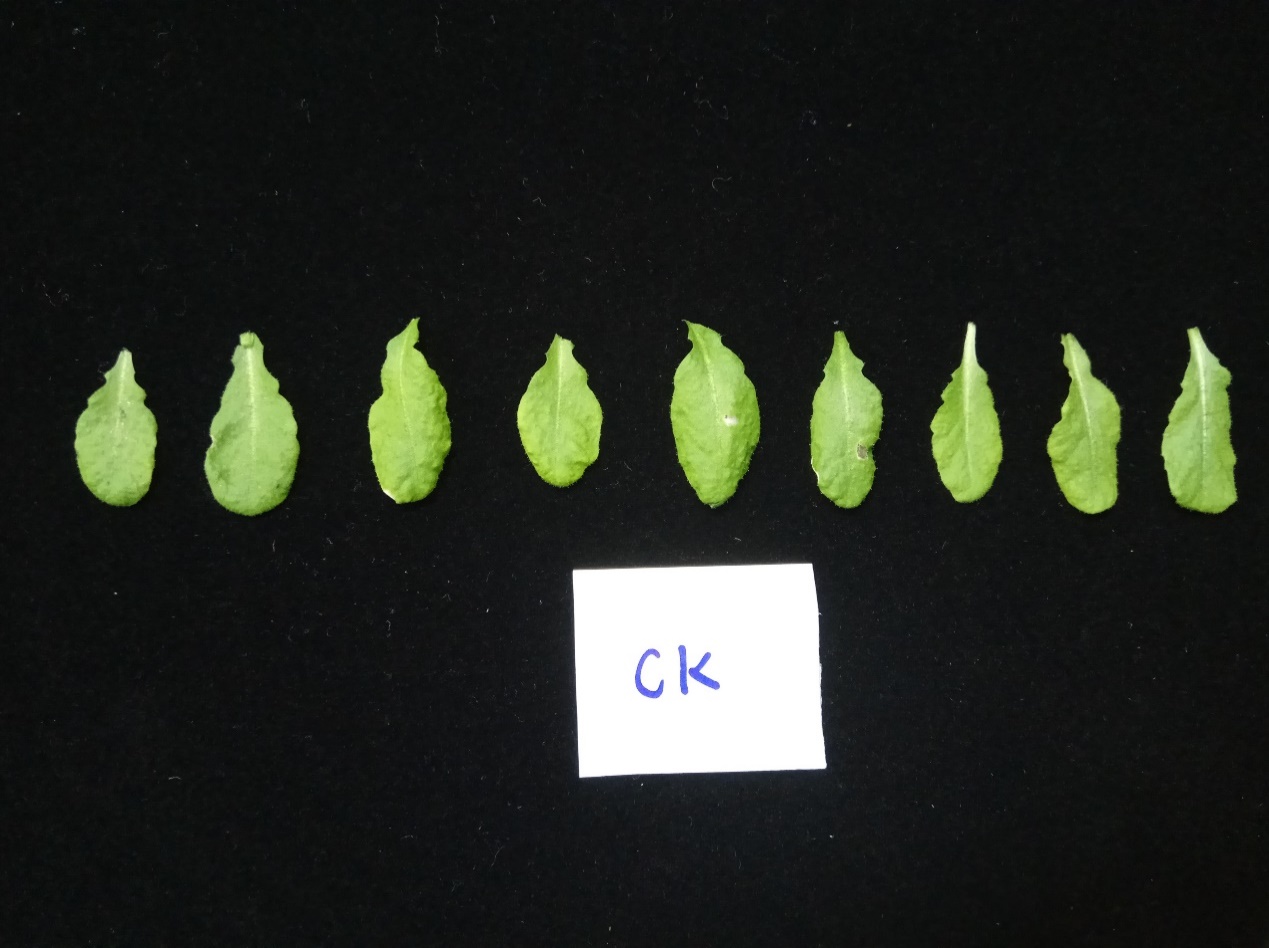


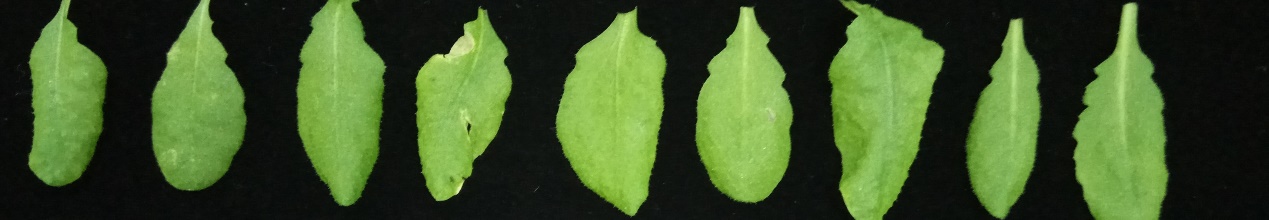

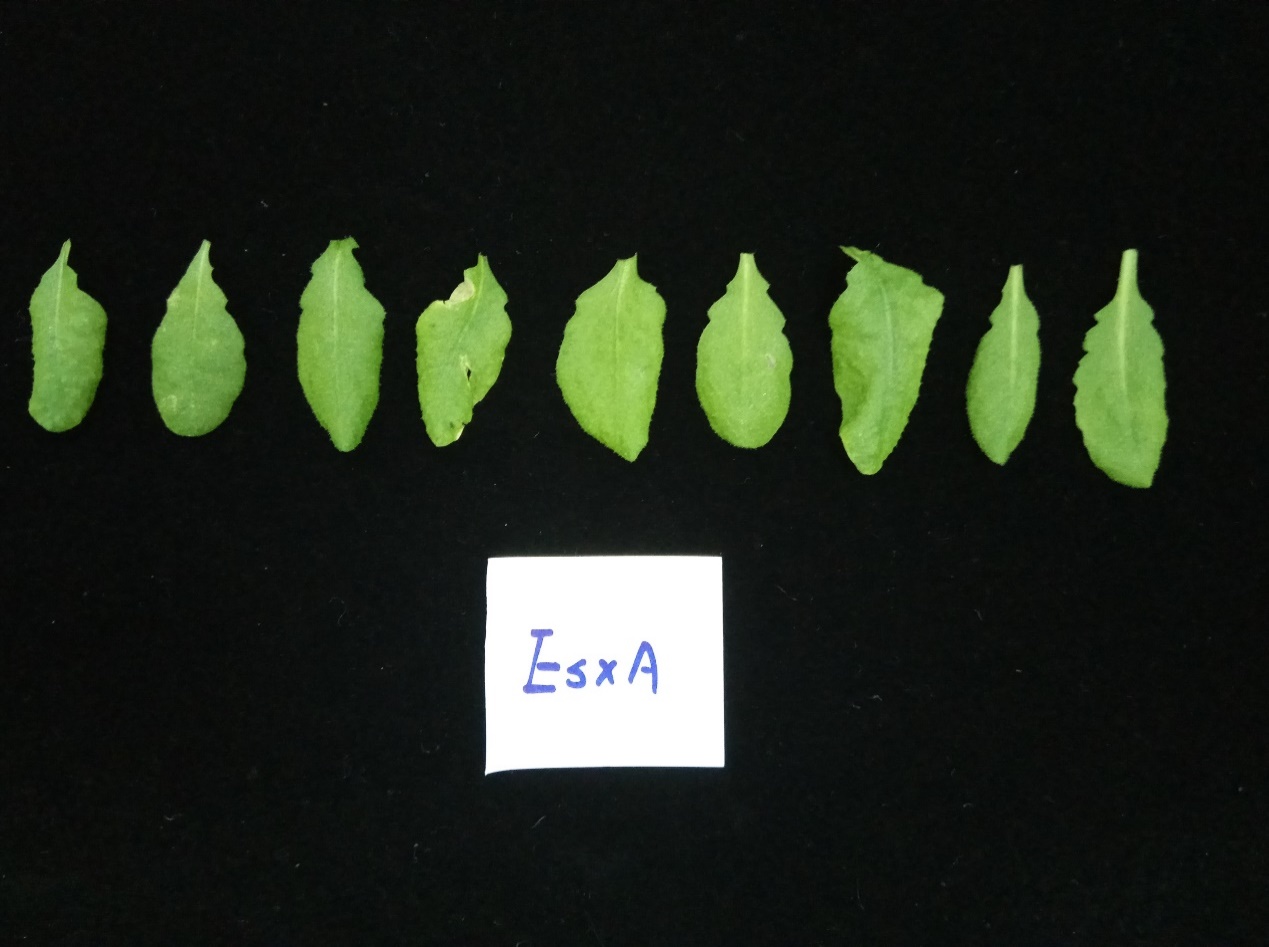

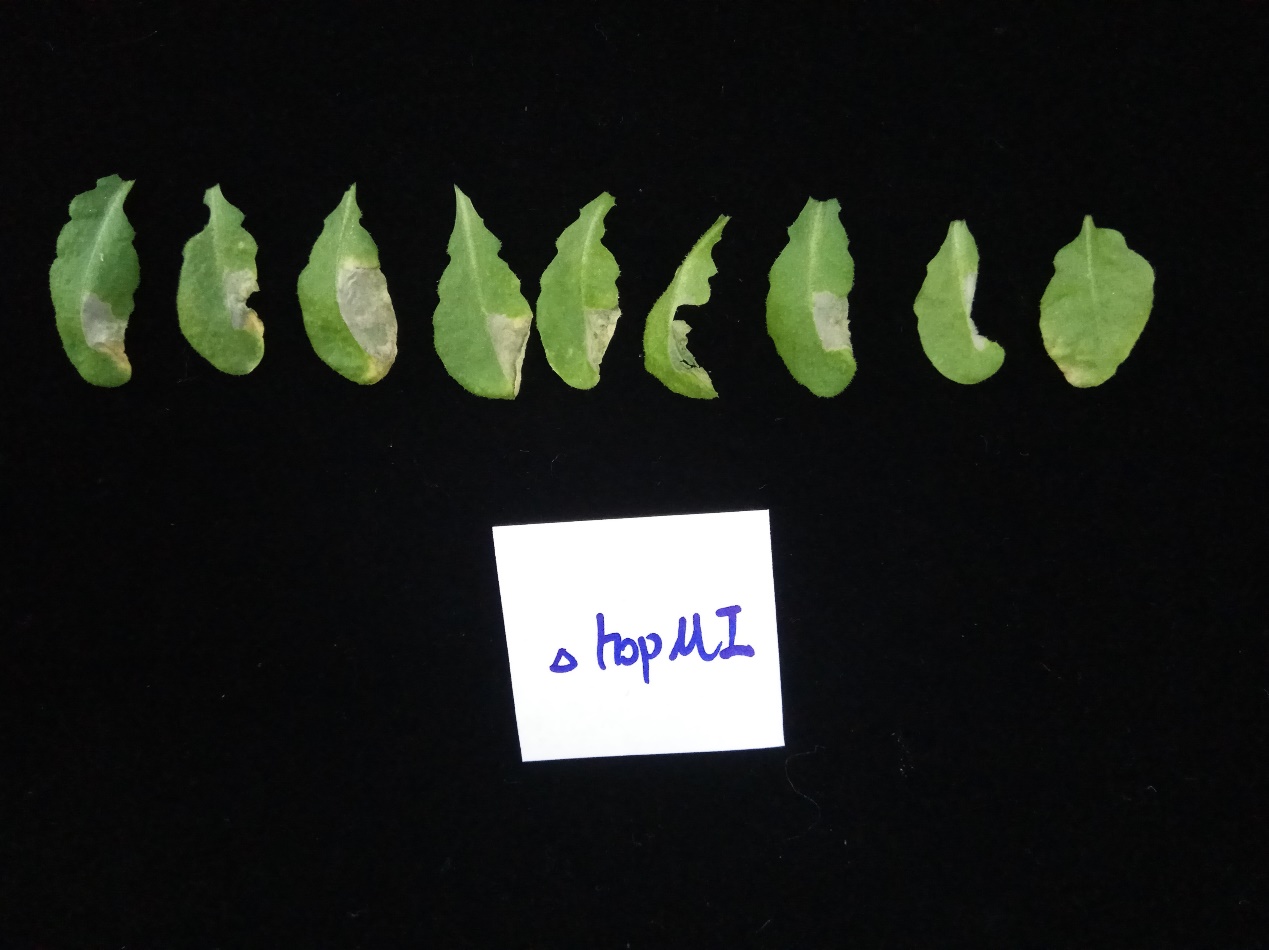

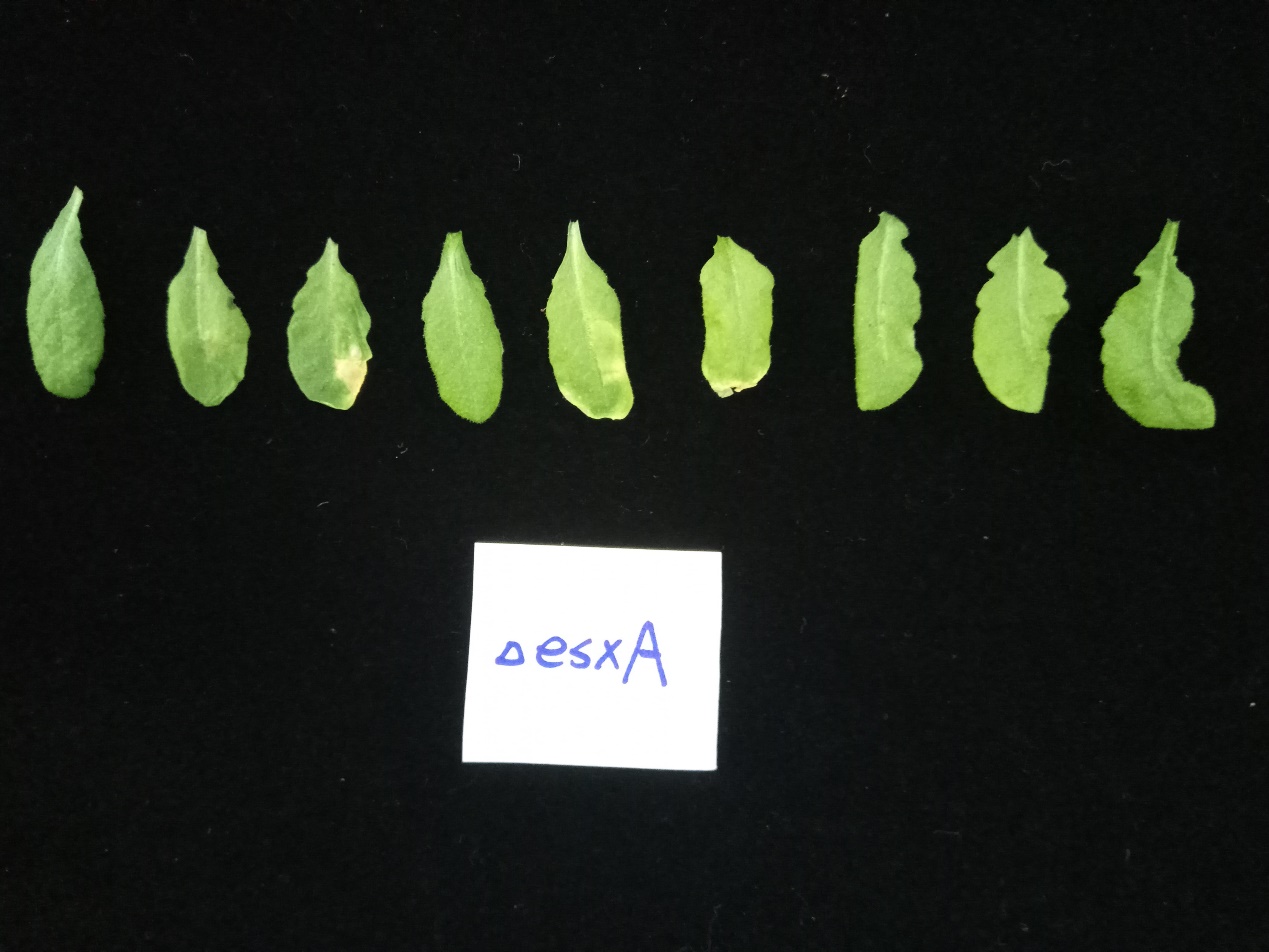


SourceData for Fig. 1B. From top to bottom, CK, D36EFLC, D36E, D36EHPM, WT.


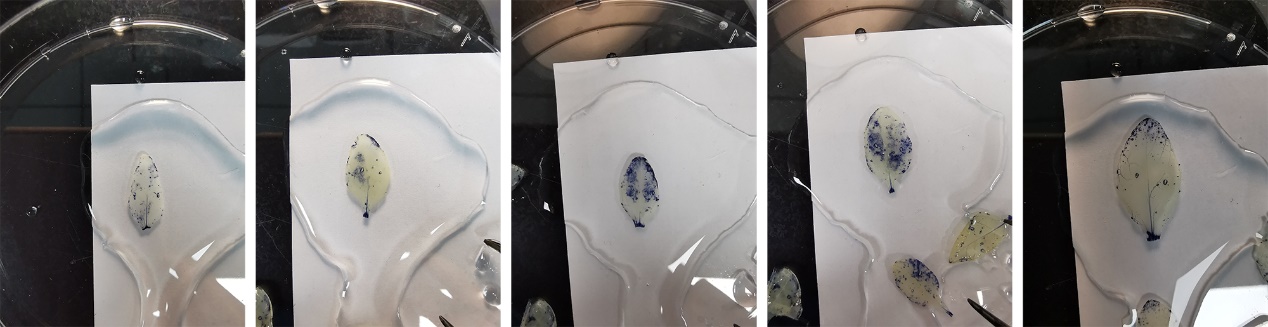


SourceData for Fig. 1D. From left to right: CK, D36EFLC, D36E, D36EHPM, WT.
